# White matter hyperintensity shape is related to long-term progression of cerebrovascular disease in community-dwelling older adults

**DOI:** 10.1177/0271678X241270538

**Published:** 2024-08-07

**Authors:** Jasmin Annica Kuhn-Keller, Sigurdur Sigurdsson, Lenore J Launer, Mark A van Buchem, Matthias JP van Osch, Vilmundur Gudnason, Jeroen de Bresser

**Affiliations:** 1 Department of Radiology, 4501 Leiden University Medical Center, Leiden, The Netherlands; 2 Icelandic Heart Association, Kopavogur, Iceland; 3 Laboratory of Epidemiology and Population Science, National Institute on Aging, Bethesda, MD, USA; 4 Faculty of Medicine, University of Iceland, Reykjavik, Iceland

**Keywords:** Cerebral small vessel disease, cerebrovascular disease, magnetic resonance imaging, vascular cognitive impairment, white matter hyperintensities

## Abstract

White matter hyperintensity (WMH) shape is associated with long-term dementia risk in community-dwelling older adults, however, the underlying structural correlates of this association are unknown. We therefore aimed to investigate the association between baseline WMH shape and cerebrovascular disease progression over time in community-dwelling older adults. The association of WMH shape and cerebrovascular disease markers was investigated using linear and logistic regression models in the Age, Gene/Environment Susceptibility-Reykjavik (AGES) study (n = 2297; average time to follow-up: 5.2 years). A more irregular shape of periventricular/confluent WMH at baseline was associated with a larger increase in WMH volume, and with occurrence of new subcortical infarcts, new microbleeds, new enlarged perivascular spaces, and new cerebellar infarcts at the 5.2-year follow-up (all p < 0.05). Furthermore, less elongated and more irregularly shaped deep WMHs were associated with a larger increase in WMH volume, and new cortical infarcts at follow-up (p < 0.05). A less elongated shape of deep WMH was associated with new microbleeds at follow-up (p < 0.05). Our findings show that WMH shape may be indicative of the type of cerebrovascular disease marker progression. This underlines the significance of WMH shape to aid in the assessment of cerebrovascular disease progression.

## Introduction

Most healthy older adults have changes on brain MRI scans related to cerebrovascular disease. The most common cerebrovascular changes are white matter hyperintensities (WMHs). These WMHs are associated with long term dementia occurrence and cognitive decline.^1,2^ However, the structural correlates of these associations remain unknown.

Different quantitative MRI markers exist to study WMH, such as the commonly used WMH volume. However, this marker can be considered rather crude and is disease-unspecific. This hinders more in-depth investigations related to disease mechanisms. In recent studies, WMH shape was introduced as a novel marker that may provide a more detailed and more disease specific characterization of WMH compared to WMH volume. Previous studies have shown that a more irregular shape of periventricular/confluent WMH is associated with the occurrence of future stroke and increased mortality in patients with an increased vascular burden.^3^ Moreover, a more irregular shape of periventricular/confluent WMHs was associated with an increased long-term risk for dementia.^4^ These studies indicate that different WMH shape patterns are probably related to different underlying pathologies (e.g. gliosis, fiber loss, and demyelination).^5^ This could provide crucial information about potential disease progression.

We hypothesized that different WMH shape patterns are related to different types of underlying pathologies and that some shape patterns may be related to progression of specific cerebrovascular markers. We therefore aimed to investigate the association between baseline WMH shape and progression of cerebrovascular disease markers over 5.2 years in community-dwelling older adults.

## Methods

### Participants & study design

**Figure 1.**
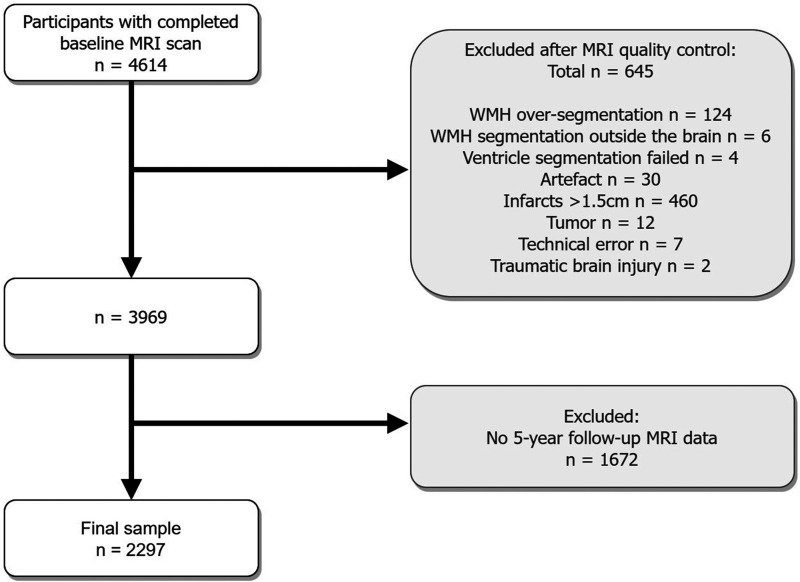
Flowchart illustrating the exclusions of the study. Participants with infarcts bigger than 1.5 cm were excluded to avoid false positive WMH segmentation. WMH oversegmentation outside the brain was automatically corrected using brain masks.

Data from the AGES Reykjavik study was used in the current study.^6^ The study was approved by the Icelandic National Bioethics Committee, VSN:00-063, and the institutional review board responsible for the National Institute on Aging (NIA) research; all participants signed informed consent. The AGES Reykjavik study was conducted in accordance with the ethical standards laid down in the 1964 Declaration of Helsinki and its later amendments. Brain MRI scans were acquired at baseline from 2002 to 2006 and approximately five years later at follow-up from 2007 to 2011. A flow-chart describing the inclusion and exclusion of participants in the current study is shown in Figure 1. Of the 4614 included participants, a total of 654 participants were excluded after MRI quality control (WMH oversegmentation: n = 124; WMH segmentation outside of the brain: n = 6; ventricle segmentation failed: n = 4); artefacts: n = 30; infarcts >1.5 cm: n = 460; tumor: n = 12; technical error: n = 7; traumatic brain injury: n = 2). WMH oversegmentation outside of the brain was automatically corrected using brain masks. Another 1672 participants were excluded because of missing follow-up data (death: n = 505 (30%), disability or refused: n = 859 (51%), lost to follow-up: n = 104 (6%), claustrophobia: n = 86 (5%), MRI contradictions: n = 116 (7%), technical issues: n = 2 (0.1%)). A total of 2297 participants were included in the current study.

### MRI acquisition protocol

The same MRI scanning protocol was used at baseline and follow-up on a 1.5 Tesla Signa Twinspeed system (General Electric Medical Systems, Waukesha, Wisconsin). Sequences included in the protocol: a 3 D T1-weighted, spoiled-gradient echo (repetition time =21 ms; time to echo = 8 ms; field of view = 240 mm; slice thickness = 1.5 mm; voxel size = 0.94 × 0.94 ×1.50 mm^3^); a fluid attenuated inversion recovery (FLAIR) (repetition time = 8000 ms; time to echo =100 ms; field of view = 220 mm; voxel size = 0.86 ×0.86 × 3.00 mm^3^); a 2 D T2*-weighted gradient-echo-type echo planar imaging (GRE-EPI) (repetition time = 3050 ms; time to echo = 50 ms; field of view: 220 mm; matrix = 256 × 256); a proton density/T2-weighted fast spin-echo (repetition time = 3220 ms; time to first echo = 22 ms; time to second echo = 90 ms; echo train length 8; field of view = 220 mm; matrix = 256 × 256). The FLAIR, T2*, and proton density/T2 scans were acquired with 3 mm thick interleaved slices with voxel sizes of 0.86 mm × 0.86 mm × 3.00 mm.

### WMH shape

**Figure 2.**
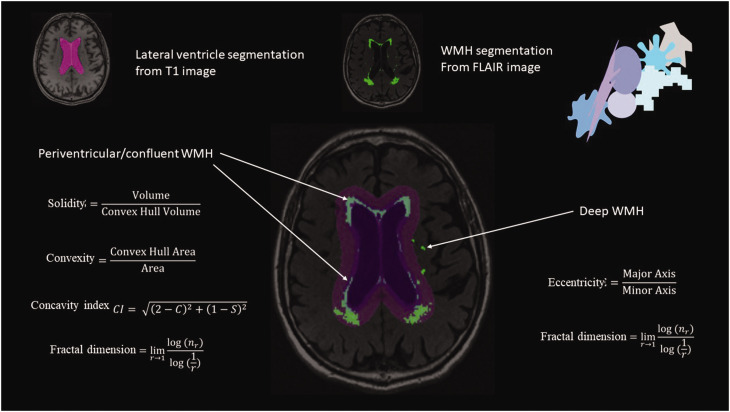
Illustration of the WMH shape image processing pipeline. Lateral ventricles were segmented using T1-weighted MRI images. WMH segmentation was performed on the registered FLAIR images. Using two different inflated ventricle masks (3 mm and 10 mm), WMH were classified into three types (deep, periventricular and confluent). Based on the resulting WMH type, different WMH shape markers were calculated using the shown formulas.

WMH shape markers were calculated using an in-house developed pipeline, as described in detail previously.^4^ In short, WMH were segmented automatically on the registered FLAIR images using the LST toolbox^7^ in SPM12. Lateral ventricles were segmented from the T1 scans and the ventricle masks were inflated with 3 and 10 mm. The inflated ventricle masks aided WMH classification into periventricular/confluent, and deep WMH (Figure 2). Periventricular and confluent WMH were merged into one category, because of spatial overlap in these lesion types preventing separated shape analyses. WMH need to be at least 3 mm distant from the ventricular wall to be classified as deep WMH. WMH shape markers were calculated based on the WMH segmentations. Convexity, solidity, concavity index, and fractal dimensions were determined for periventricular/confluent WMHs.^8^ A lower convexity and solidity, and higher concavity index and fractal dimension indicate more irregularly shaped periventricular/confluent WMH. Fractal dimensions and eccentricity were calculated for deep WMH.^8^ A higher eccentricity indicates a more elongated shape, while a higher fractal dimensions indicates a more irregular shape of deep WMH. The formulas used to calculate the WMH shape markers are shown in Figure 2. Average shape markers were calculated per participant. Each WMH shape marker captures a slightly different variation of WMH shape. In supplementary figure 1 examples of shapes with high or low values of different shape markers are shown.

### Other cerebrovascular markers

Gray matter, white matter, cerebrospinal fluid and WMH volume were segmented automatically with a modified algorithm based on the Montreal Neurological Institute pipeline.^9^ Intracranial volume resulted from the addition of the volumes of gray matter, white matter, cerebrospinal fluid and WMH.^10^ The same processing-pipeline was used at baseline and follow-up. The WMH volume change was calculated by substracting the baseline WMH volumes from the follow-up WMH volumes.

Occurrence of new subcortical brain infarcts, microbleeds, enlarged perivascular spaces, cerebellar infarcts, and cortical infarcts at follow-up were rated by trained radiographers by comparing the baseline and follow-up MRI scans.^11^ Follow-up MRI scans were examined and if a lesion was detected, the baseline scan was checked to see if the lesion was new.

Microbleeds were scored if they were visible on the T2*-weighted scans.^12^ Infarcts were scored if they were visible on the FLAIR, T2-weighted and the proton-density scan.^13^ Cortical infarcts were labelled as such if they involved or were limited to the cerebral cortex and were surrounded by an area of high signal intensity on FLAIR images. Cerebellar infarcts had no size criteria. Subcortical infarcts were scored if they did not extend into the cortex and were surrounded by an area of high signal intensity on FLAIR images with a minimal diameter of 4 mm.^13^ Enlarged perivascular spaces were defined as round or tubular defects with a short axis >3 mm in the subcortical area, without a surrounding area with high signal intensity on FLAIR images.^14^

### Baseline characteristics and cardiovascular risk factors

Baseline information, such as age, sex, education level, and smoking status, were collected via questionnaires. The highest completed education level (primary school, secondary school, college, or university) was noted. Non-smokers were participants who never smoked, former smokers were regular smokers of at least 100 cigarettes or 20 cigars in a lifetime, and the third category was current smokers. Participant’s height (cm) and weight (kg) were measured and used to calculate body mass index (BMI). Systolic and diastolic blood pressure were measured using a standard mercury sphygmomanometer; the mean of 2 measurements was calculated. Hypertension was registered in the database based on self-report and/or use of antihypertensive medication, and/or measured systolic blood pressure >140 mm Hg and/or diastolic blood pressure >90 mm Hg. Diabetes mellitus was registered based on self-report of diabetes, or use of anti-diabetic medication, or fasting blood glucose level >7.0 mmol/L. Coronary artery disease was registered upon self-report and the use of nitrates, a coronary bypass, and/or evidence of myocardial infarction on an electrocardiogram.

### Statistical analysis

Solidity, convexity, and eccentricity were inverted for the logistic regression analyses to aid comparability of the results. Solidity and baseline WMH volumes were multiplied by 100 and natural log transformed due to non-normal distribution. Z-scores of WMH shape markers and baseline WMH volumes were calculated to aid comparability. The average intracranial volume of baseline and follow-up was calculated to improve precision. To study the association between periventricular/confluent and deep WMH shape markers and change in WMH volume, linear regression analyses controlled for age, sex, and intracranial volume were performed. To study the association between periventricular/confluent and deep WMH shape markers and occurrence of new subcortical brain infarcts, microbleeds, enlarged perivascular spaces, cerebellar infarcts, and cortical infarcts, logistic regression analyses were performed controlled for age and sex.

As a frame of reference, the association between baseline WMH volume and change in WMH volume was tested with linear regression analyses controlled for age, sex, and intracranial volume. Furthermore, the association between baseline WMH volume and occurrence of new subcortical brain infarcts, microbleeds, enlarged perivascular spaces, cerebellar infarcts, and cortical infarcts was tested with logistic regression analyses controlled for age, and sex. A p value < 0.05 was considered statistically significant.

## Results

A total of 2297 participants were included in the current study. A flowchart showing the inclusions/exclusions of the study is shown in Figure 1. Supplementary table 1 contains a comparison of the baseline characteristics of the participants included (n = 2297) and excluded (n = 2317) from our study. As to be expected, cardiovascular risk factors, such as diabetes, coronary artery disease, and hypertension were significantly more prevalent in the excluded group. Moreover, the excluded group is older and has a lower BMI.

The mean age at baseline of the included community-dwelling older adults was 74.5 ± 4.7 years and the mean time to follow-up was 5.2 ± 0.2 years. The characteristics of the study sample, including cardiovascular risk factors, are shown in Table 1. At baseline 153 participants (7%) had subcortical infarcts, 381 participants (17%) had microbleeds, 358 participants (16%) had enlarged perivascular spaces, 428 participants (19%) had cerebellar infarcts, and 176 participants (8%) had cortical infarcts (Table 2). The average WMH volume at baseline was 16.6 ± 17.2 ml. At follow-up, 68 participants (3%) had new subcortical infarcts, 299 participants (13%) had new microbleeds, 39 participants (2%) had new enlarged perivascular spaces, 168 participants (7%) had new cerebellar infarcts and 123 participants (5%) had new cortical infarcts (Table 2). The average WMH volume at follow-up was 22.3 ± 22.2 ml. A detailed description of the cerebrovascular changes on MRI of the participants can be found in supplementary table 2.

**Table 1.** Characteristics of the participants.

	Older adult participants (n = 2297)
Age at baseline (years)	74.5 ± 4.7
Females	1399 (61%)
Hypertension	1763 (77%)
Type 2 diabetes mellitus	192 (8%)
BMI at baseline (kg/m^3^)	27.2 ± 4.1
Cholesterol (mmol/L)	5.70 ± 1.13
Smoking status	
Never	1017 (44%)
Former	1030 (45%)
Current	250 (11%)
Coronary artery disease	361 (16%)
Time to follow-up (years)	5.2 ± 0.2

Data are shown as mean ± SD or frequency (%). Baseline characteristics were collected via questionnaires.

**Table 2.** Cerebrovascular MRI markers of the participants.

	Baseline	Follow-up	Change over time
Periventricular/confluent WMH			
Solidity	0.19 ± 0.12	–	–
Convexity	1.03 ± 0.18	–	–
Concavity index	1.27 ± 0.15	–	–
Fractal dimension	1.71 ± 0.15	–	–
Deep WMH		–	–
Eccentricity	0.61 ± 0.07	–	–
Fractal dimension	1.70 ± 0.14	–	–
WMH volume (ml)	16.56 ± 17.21	22.32 ± 22.18	5.76 ± 7.74
Participants with subcortical infarcts	153 (7%)	221 (10%)	68 (3%)
Participants with microbleeds	381 (17%)	680 (30%)	299 (13%)
Participants with enlarged perivascular spaces	358 (16%)	397 (17%)	39 (2%)
Participants with cerebellar infarcts	434 (19%)	596 (26%)	162 (7%)
Participants with cortical infarcts	176 (8%)	299 (13%)	123 (5%)

Data are shown as mean ± SD, or frequency (%).

### Periventricular/confluent WMH shape and progression of cerebrovascular changes

A more irregular shape of periventricular/confluent WMH (lower solidity (B: 0.91 (95% CI: 0.59–1.23); p < 0.001), lower convexity (B: 1.94 (1.62–2.26); p < 0.001), higher concavity index (B: 2.28 (1.95–2.61); p < 0.001), higher fractal dimension (B: 2.62 (2.31–2.93); p < 0.001)) at baseline was significantly associated with a larger increase in WMH volume over 5.2 years (Table 3 and supplementary figure 2). Furthermore, a more irregular shape of periventricular/confluent WMH at baseline was associated with new subcortical infarcts (lower solidity (OR: 1.75 (95% CI: 1.16–2.62); p < 0.001); lower convexity (OR: 1.44 (1.17–1.76); p < 0.001); higher concavity index (OR: 1.58 (1.29–1.94); p < 0.001); higher fractal dimension (OR: 1.91 (1.49–2.44); p < 0.001)), new microbleeds (lower solidity (OR: 1.24 (1.07–1.44); p = 0.004); lower convexity (OR: 1.16 (1.04–1.30); p = 0.009); higher concavity index (OR: 1.24 (1.11–1.39); p < 0.001); higher fractal dimension (OR: 1.47 (1.30–1.65); p < 0.001)), new enlarged perivascular spaces (lower convexity (OR: 1.34 (1.05–1.71); p < 0.017); higher concavity index (OR: 1.34 (1.05–1.71); p < 0.020)), and new cerebellar infarcts at follow-up (lower convexity (OR: 1.16 (1.02–1.33); p < 0.022); higher concavity index (OR: 1.16 (1.02–1.33); p < 0.027)). Periventricular/confluent WMH shape at baseline was not significantly associated with new cortical infarcts at follow-up (solidity (OR: 1.27 (0.97–1.65); p = 0.078), convexity (OR: 1.04 (0.88–1.22); p = 0.652), concavity index (OR: 1.09 (0.92–1.29); p = 0.345), fractal dimension (OR: 1.20 (1.00–1.44); p = 0.051).

**Table 3.** Linear and logistic regression analyses of baseline WMH shape and volume markers and cerebrovascular changes over 5.2 years.

	Change in WMH volumeB (95% CI)	New subcortical infarctsOR (95% CI)	New microbleedsOR (95% CI)	New enlarged perivascular spacesOR (95% CI)	New cerebellar infarctsOR (95% CI)	New cortical infarctsOR (95% CI)
Periventricular/confluent WMH (n = 2297)				
Solidity	0.91 (0.59–1.23)***	1.75 (1.16–2.62)**	1.24 (1.07–1.44)**	1.07 (0.79–1.47)	0.99 (0.86–1.15)	1.27 (0.97–1.65)
Convexity	1.94 (1.62–2.26)***	1.44 (1.17–1.76)***	1.16 (1.04–1.30)**	1.34 (1.05–1.71)*	1.16 (1.02–1.33)*	1.04 (0.88–1.22)
Concavity index	2.28 (1.95–2.61)***	1.58 (1.29–1.94)***	1.24 (1.11–1.39)***	1.34 (1.05–1.71)*	1.16 (1.02–1.33)*	1.09 (0.92–1.29)
Fractal dimension	2.62 (2.32–2.93)***	1.91 (1.49–2.44)***	1.47 (1.30–1.65)***	1.28 (0.97–1.68)	1.14 (0.99–1.31)	1.20 (1.00–1.44)
Deep WMH (n = 2293)				
Eccentricity	2.02 (1.72–2.33)***	1.04 (0.84–1.28)	1.14 (1.01–1.27)*	0.97 (0.75–1.26)	1.02 (0.89–1.17)	1.30 (1.10–1.55)**
Fractal dimension	2.09 (1.78–2.41)***	1.09 (0.88–1.35)	1.03 (0.92–1.15)	1.04 (0.81–1.34)	1.06 (0.93–1.21)	1.31 (1.11–1.55)**
WMH volume (n = 2297)	4.05 (3.77–4.33)***	2.26 (1.75–2.92)***	1.52 (1.35–1.70)***	1.28 (0.99–1.64)	1.25 (1.09–1.42)**	1.44 (1.21–1.72)***

Lower solidity and convexity, as well as a higher concavity index and fractal dimension indicate a more irregular WMH shape. Higher eccentricity indicates a rounder shape and a lower eccentricity indicates a more elongated shape. Solidity, convexity, and eccentricity were inverted to aid comparability in direction of effect. The association of baseline WMH shape with WMH volume change was investigated using linear regression models, controlled for age, sex, and intracranial volume. The association of baseline WMH shape with new subcortical brain infarcts, microbleeds, enlarged perivascular spaces, cerebellar infarcts, and cortical infarcts at follow-up was investigated with logistic regression models, controlled for age and sex. Data is shown as B values (95% CI) or odds ratio (OR) (95% CI). Positive B values indicate that WMH progression increases with every unit increase of the WMH shape marker. Negative B values show that WMH progression decreases with every unit increase of the WMH shape marker. OR above 1 show that with increase of the WMH shape marker, the risk is OR-times higher to have e.g. an infarct. OR below 1 show that with increase of the WMH shape marker, the risk is OR-times lower to have e.g. infarcts. **p < 0.01 ***p < 0.001.

### Deep WMH shape and progression of cerebrovascular changes

A less elongated and irregular shape of deep WMH at baseline was significantly associated with a larger increase in WMH volume (lower eccentricity (B: 2.02 (1.72–2.33); p < 0.001); higher fractal dimension (B: 2.09 (1.78–2.41); p < 0.001)), and with new cortical infarcts at follow-up (lower eccentricity (OR: 1.30 (1.10–1.55); p < 0.003); higher fractal dimension (OR: 1.31 (1.11–1.55); p = 0.001)) (Table 3 and supplementary figures 2 and 7). Furthermore, a less elongated shape of deep WMH was associated with new microbleeds at follow-up (lower eccentricity: OR:1.14 (1.01–1.27); p = 0.027). Baseline deep WMH shape markers were not significantly associated with new subcortical infarcts, new enlarged perivascular spaces, or new cerebellar infarcts at follow-up. WMH shape markers in participants with or without cerebrovascular changes can be found in supplementary figure 3 to 7.

### WMH volume and progression of cerebrovascular changes

A higher baseline WMH volume was significantly associated with a larger increase in WMH volume over 5.2 years (B: 4.05 (3.77–4.33); p < 0.001); Table 3). Moreover, a higher baseline WMH volume was associated with new subcortical infarcts (OR: 2.26 (1.75–2.92); p < 0.001), new microbleeds (OR: 1.52 (1.35–1.70); p < 0.001), new cerebellar infarcts (OR: 1.25 (1.09–1.42); p < 0.001), and new cortical infarcts at follow-up (OR: 1.44 (1.21–1.72); p < 0.001), but not with new enlarged perivascular spaces at follow-up (OR: 1.28 (0.99–1.64); p = 0.058).

## Discussion

We found that a more irregular shape of periventricular/confluent WMH at baseline was associated with a larger increase in WMH volume, and with occurrence of new subcortical infarcts, new microbleeds, new enlarged perivascular spaces, and new cerebellar infarcts at the 5.2 year follow-up. Furthermore, less elongated and more irregularly shaped deep WMHs were associated with a larger increase in WMH volume, and new cortical infarcts at follow-up. A rounder shape of deep WMH was associated with new microbleeds at follow-up.

It has been previously shown that a more irregular WMH shape is associated with an increased long-term risk for dementia in community-dwelling older adults,^4^ but how this effect is mediated remained unclear. The current study showed that specific WMH shape patterns are indicative of specific markers of cerebrovascular disease progression.

The aetiology of WMH of presumed vascular origin in older adults is heterogenous and the exact pathophysiology remains poorly understood. Based upon our findings, we can speculate that a more irregular shape of WMH may reflect a more severe underlying aetiology of cerebrovascular disease, and as such is subsequently followed by increased progression of cerebrovascular disease-related brain changes. Histopathological studies have previously suggested that a more irregular WMH shape is associated with more severe parenchymal damage.^5,15^ Areas of smooth periventricular WMH showed demyelination and tortuous venules, as well as damage to the ventricular lining.^5^ On the other hand, previous histopathological investigations showed that irregular periventricular/confluent WMH showed gliosis, fiber loss, and reduced myelin.^5^ Moreover, the vessel walls in the areas of irregular WMH were thickened due to fibrohyalinosis and lipohyalinosis.^5^ A more complex and irregular shape of WMH may, therefore, be related to underlying pathologies that accelerate cerebrovascular disease progression.

WMH are a hallmark imaging marker of cerebral small vessel disease (SVD). In SVD changes in the normal functioning of especially the arterioles, and capillaries (such as small or large vessel atheromas or emboli) subsequently lead to WMH through hypoperfusion. MRI markers that are typically associated with SVD are WMHs, microbleeds, enlarged perivascular spaces and subcortical infarcts.^16^ Large vessel disease (LVD), on the other hand, is mostly caused by atherosclerosis and atheroma depositions in the wall of larger upstream arteries, such as the carotid arteries. A typical brain MRI marker of LVD is a cortical infarct.^16^ LVD can also lead to lacune-like infarcts via atheromas in the larger parent arteries,^16,17^ which is also where SVD and LVD pathology may be linked.^16^

There are several possible mechanisms to explain the association of WMH shape and progression of different SVD and LVD markers. For example, subcortical infarcts and WMHs may share (part of) the same SVD-related pathological pathways.^18,19^ WMHs might be related to subcortical infarcts via secondary hypoperfusion and the resulting ischemia in the parenchyma surrounding the WMHs.^20^ We found that a more irregular shape of periventricular/confluent WMHs was related to, among others, new subcortical infarcts, new microbleeds, and new enlarged perivascular spaces. In a previous population-based study, pre-existing and incident microbleeds were associated with incident lacunes and progression of WMH volume.^21^ The authors propose that this association can be explained by shared pathways of haemorrhagic and ischemic pathologies in the preclinical phase of cerebrovascular disease.^21^ While some previous studies report an association between WMH burden and enlarged perivascular spaces, a previous meta-analysis has concluded that there is no clear association.^22^ In the current study, baseline periventricular/confluent WMH shape was associated with new enlarged perivascular spaces at follow-up, but we did not find an association between baseline WMH volume and new enlarged perivascular spaces at follow-up. This result was found despite the relatively limited progression of enlarged perivascular spaces at follow-up. This suggests that WMH shape might contain additionally relevant information regarding SVD compared to WMH volume alone. Furthermore, enlarged perivascular spaces are a relatively new marker of SVD^23^ and the current findings strengthen their presumed connection to SVD pathology. Combined, these findings illustrate that WMH shape may help to differentiate between different further subtypes of SVD, which is reflected in the association of different WMH shape patterns to different cerebrovascular markers. For example previous research has already shown that different more rare subtypes of SVD lead to different patterns of MRI changes (e.g. in cerebral amyloid angiopathy (CAA) or cerebral autosomal dominant arteriopathy with subcortical infarcts and leukoencephalopathy (CADASIL)^24,25^), but much is still unknown in this heterogenous disease. WMH shape may help to further differentiate subtypes of SVD by providing a more detailed characterization of WMHs on MRI.

In the current study, less elongated and more irregularly shaped deep WMHs were associated, among others, with new cortical infarcts at follow-up. Since cortical infarcts are a marker of LVD, the related WMH shape patterns of deep WMH may be indicative of a pathophysiological involvement of LVD. Periventricular/confluent WMH shape, on the other hand, was not related to new cortical infarcts at follow-up. Cerebellar infarcts can represent both SVD and LVD and are presumed to be of embolic origin. In the current study, cerebellar infarcts were associated with periventricular/confluent WMH shape, but not with deep WMH shape markers. The difference of the results depending on WMH type might support the notion that deep and periventricular/confluent WMH are resulting from different underlying pathologies.^26,27^ Different baseline WMH shape patterns were associated with progression of different cerebrovascular disease markers at the 5-year follow-up, which shows that WMH shape might be indicative of the type of cerebrovascular disease progression at an early stage.

The strengths of the current study are the automatic assessment of WMH shape and volume, and the longitudinal study design with a relatively long follow-up. Significant external validity is added to the study by the large sample size from the general population. A limitation of the current study is the use of a 1.5 T MRI system, which was a common field strength for clinical MRI scanners at the time of data collection. MRI images with a lower signal-to-noise ratio or spatial resolution could have resulted in a less accurate WMH shape estimation. However, we have reported on significant associations with cerebrovascular disease markers despite this limitation. Another limitation of our study is that lacunes were not scored separately, but were scored in the category of subcortical infarcts. However, we expect the possible effect of this to be small as the prevalence of large subcortical infarct is very low. Another limitation of our study could be that the strength of the associations found in the current study may have been influenced and weakened by selective loss to follow-up. It is likely that participants who had died, refused or were not eligible for a follow-up MRI were less healthy than the participants who did undergo the follow-up. This could have resulted in an underestimation of the associations shown in our study.

In conclusion, our findings show that WMH shape may be indicative of the type of cerebrovascular disease marker progression. This underlines the significance of WMH shape to aid in the assessment of cerebrovascular disease progression.

## Supplemental Material

sj-pdf-1-jcb-10.1177_0271678X241270538 - Supplemental material for White matter hyperintensity shape is related to long-term progression of cerebrovascular disease in community-dwelling older adults Supplemental material, sj-pdf-1-jcb-10.1177_0271678X241270538 for White matter hyperintensity shape is related to long-term progression of cerebrovascular disease in community-dwelling older adults by Jasmin Annica Kuhn-Keller, Sigurdur Sigurdsson, Lenore J Launer, Mark A van Buchem, Matthias JP van Osch, Vilmundur Gudnason and Jeroen de Bresser in Journal of Cerebral Blood Flow & Metabolism
