# Supplementary material for: White matter hyperintensity shape is related to long-term progression of cerebrovascular disease in community-dwelling older adults
Source: J Cereb Blood Flow Metab. 2024 Aug 7;45(1):187–95. doi: 10.1177/0271678X241270538 (PMC11572234; doi:10.1177/0271678X241270538)
Supplement: sj-pdf-1-jcb-10.1177_0271678X241270538 - Supplemental material for White matter hyperintensity shape is related to long-term progression of cerebrovascular disease in community-dwelling older adults [file sj-pdf-1-jcb-10.1177_0271678X241270538.pdf]

Supplementary Material

Supplementary table 1. Baseline characteristics of the participants included and excluded from our study.

	Included participants (n= 2297)	*Excluded participants (n= 2317)*	*p-values*
Age at baseline (years)	74.5 ± 4.7	78.3 ± 5.6	<0.001
Females	1399 (61%)	1281 (55%)	<0.001
Hypertension	1763 (77%)	2226 (96%)	<0.001
Type 2 diabetes mellitus	192 (8%)	324 (14%)	<0.001
BMI at baseline (kg/m^3)	27.2 ± 4.1	26.8 ± 4.6	<0.001
Cholesterol (mmol/L)	5.70 ± 1.13	5.58 ± 1.18	<0.001
Smoking status			
Never	1017 (44%)	979 (42%)	0.166
Former	1030 (45%)	1031 (45%)	0.178
Current	250 (11%)	304 (13%)	0.019
Coronary artery disease	361 (16%)	760 (33%)	<0.001

Data are shown as mean ± SD or frequency (%). Baseline characteristics were collected via questionnaires. One-way ANOVA’s were performed for continuous variables, and Chi-square tests for categorical variables.

Supplementary table 2. In depth cerebrovascular MRI markers of the participants (n=2297).

	Baseline	***Follow-up***	***Change over time***
WMH volume (ml)	16.56 ± 17.21	22.32 ± 22.18	5.76 ± 7.74
Participants with subcortical infarcts	153 (7%)	221 (10%)	68 (3%)
with 1 subcortical infarct	118 [77%]	157 [71%]	39 [57%]
with 2 subcortical infarcts	21 [14%]	33 [15%]	12 [18%]
with 3 subcortical infarcts	5 [3%]	17 [8%]	12 [18%]
with 4 subcortical infarcts	5 [3%]	7 [3%]	2 [3%]
with ≥5 subcortical infarcts	4 [3%]	7 [3%]	3 [4%]
Participants with microbleeds	381 (17%)	680 (30%)	299 (13%)
with 1 microbleed	268 [70%]	434 [64%]	166 [56%]
with 2 microbleeds	62 [16%]	119 [18%]	57 [19%]
with 3 microbleeds	22 [6%]	46 [7%]	24 [8%]
with 4 microbleeds	14 [4%]	27 [4%]	13 [4%]
with ≥5 microbleeds	15 [4%]	54 [8%]	39 [13%]
Participants with enlarged PVS	358 (16%)	397 (17%)	39 (2%)
with 1 enlarged PVS	273 [76%]	296 [75%]	23 [59%]
with 2 enlarged PVS	54 [15%]	62 [16%]	8 [21%]
with 3 enlarged PVS	13 [4%]	20 [5%]	7 [18%]
with 4 enlarged PVS	10 [3%]	7 [18%]	-3 [8%]
with ≥5 enlarged PVS	8 [2%]	12 [3%]	4 [10%]
Participants with cerebellar infarcts	434 (19%)	596 (26%)	162 (7%)
with 1 cerebellar infarct	279 [64%]	336 [56%]	57 [35%]
with 2 cerebellar infarcts	91 [22%]	140 [23%]	43 [27%]
with 3 cerebellar infarcts	19 [4%]	50 [8%]	31 [19%]

with 4 cerebellar infarcts	14 [3%]	22 [4%]	8 [5%]
with ≥5 cerebellar infarcts	25 [6%]	48 [8%]	23 [14%]
Participants with of cortical infarcts	176 (8%)	299 (13%)	123 (5%)
with 1 cortical infarct	134 [76%]	189 [63%]	55 [45%]
with 2 cortical infarcts	28 [16%]	66 [22%]	38 [31%]
with 3 cortical infarcts	10 [6%]	22 [7%]	12 [10%]
with 4 cortical infarcts	1 [1%]	7 [2%]	6 [5%]
with ≥5 cortical infarcts	3 [2%]	15 [5%]	12 [10%]

Data are shown as mean ± SD, or frequency (%). The table shows number of participants with cerebrovascular disease markers and a specification in markers. Number of participants with cerebrovascular disease marker as percentage of the total sample size (in brackets: ( )). Number of participants with a specific cerebrovascular disease markers as percentage of the total number of participants with this cerebrovascular disease marker (in square brackets: [ ]). PVS: perivascular spaces.

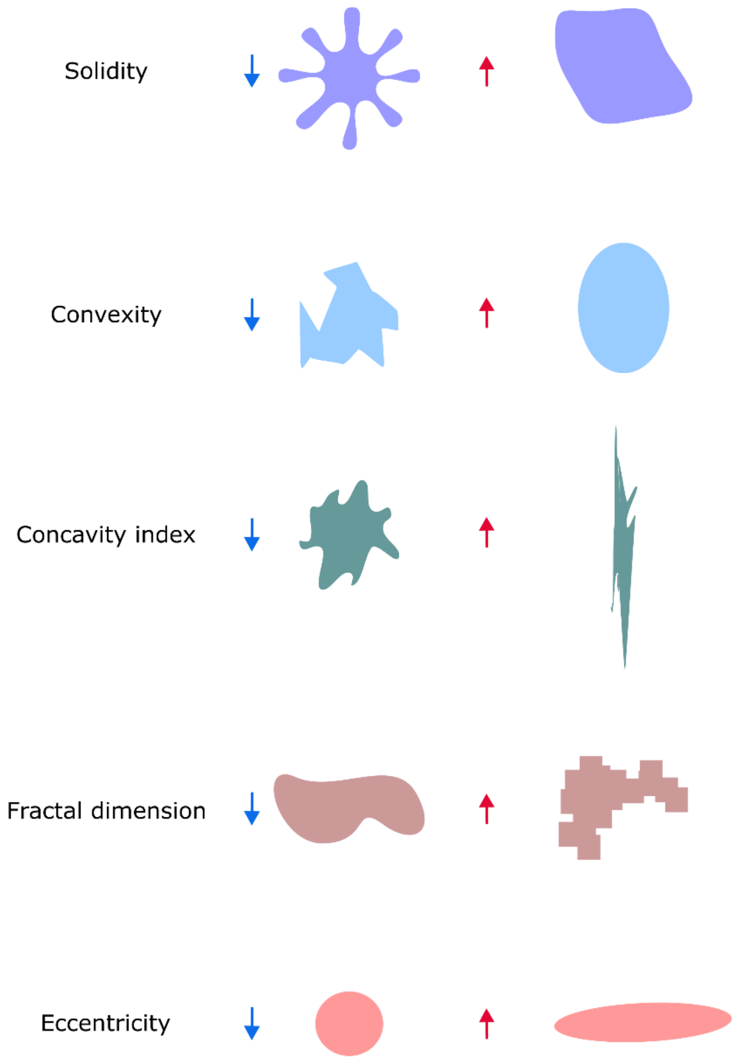

Supplementary figure 1. WMH shape markers and examples of shapes with high or low values of different shape markers. For periventricular/confluent WMHs solidity, convexity, concavity index, and fractal dimension were calculated. For deep WMHs eccentricity and fractal dimension were the calculated shape markers.

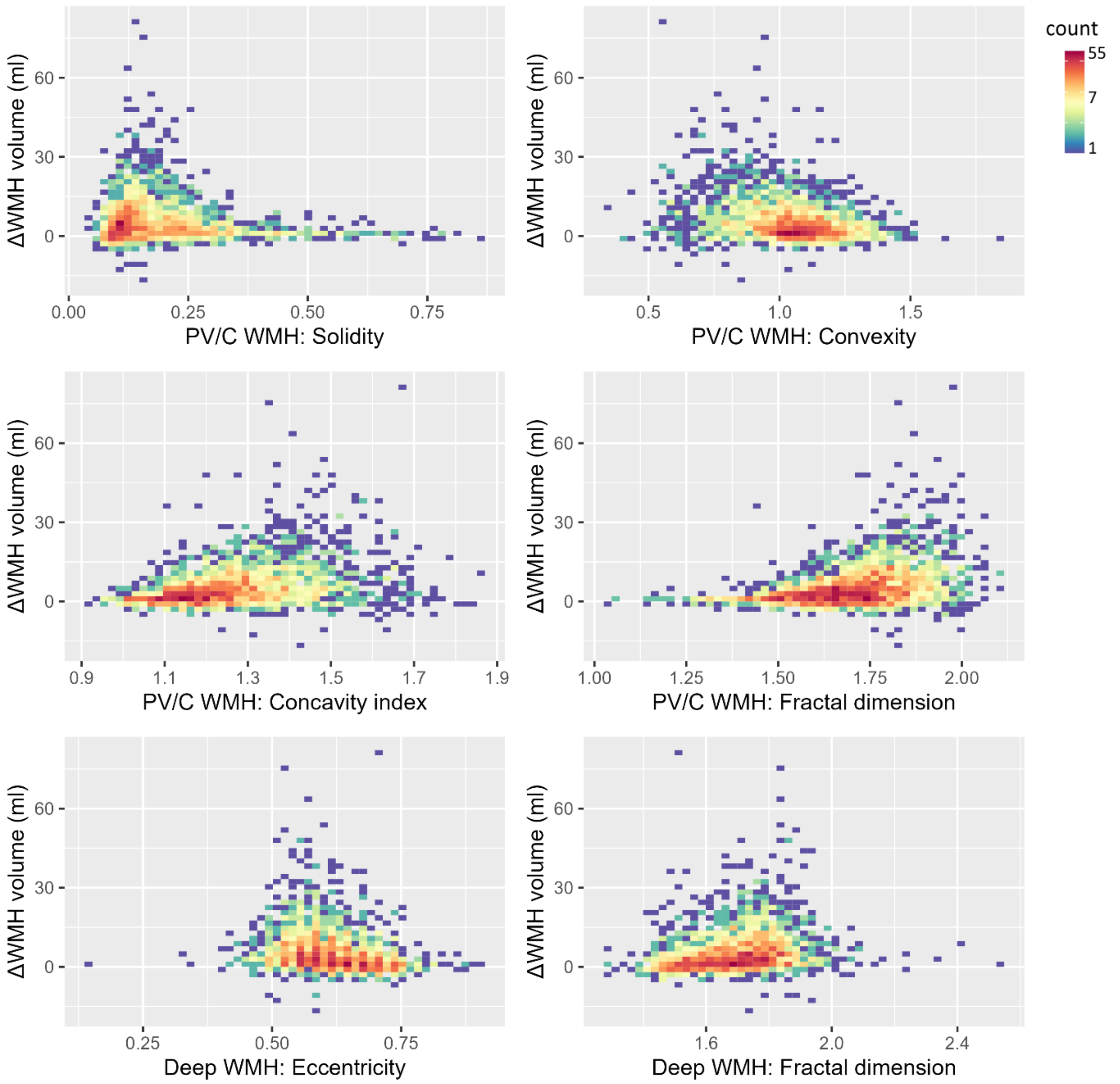

Supplementary figure 2. Density plots showing baseline WMH shape markers in relation to change in WMH volume over time determined at the 5-year follow-up. The color scale represents the number of participants per datapoint.

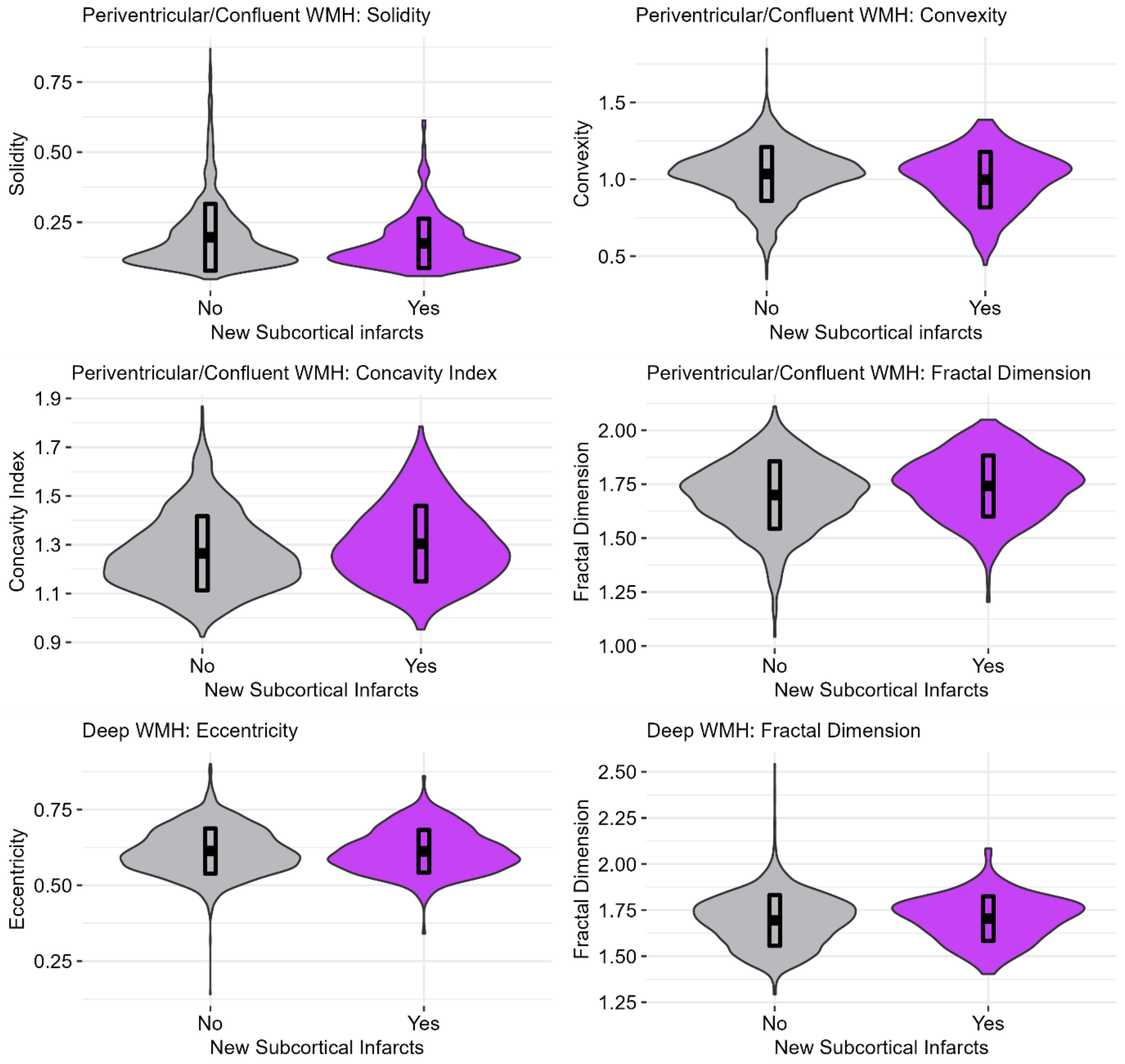

Supplementary figure 3. WMH shape markers at baseline in participants with new subcortical infarcts versus participants without new subcortical infarcts at follow-up. A more irregular shape of periventricular/confluent WMH at baseline was associated with new subcortical infarcts (lower solidity (OR: 1.75 (95% CI: 1.16–2.62); $p<0.001$); lower convexity (OR: 1.44 (1.17–1.76); $p<0.001$); higher concavity index (OR: 1.58 (1.29–1.94); $p<0.001$); higher fractal dimension (OR: 1.91 (1.49–2.44); $p<0.001$)). We did not find an association of deep WMH shape markers and new subcortical infarcts.

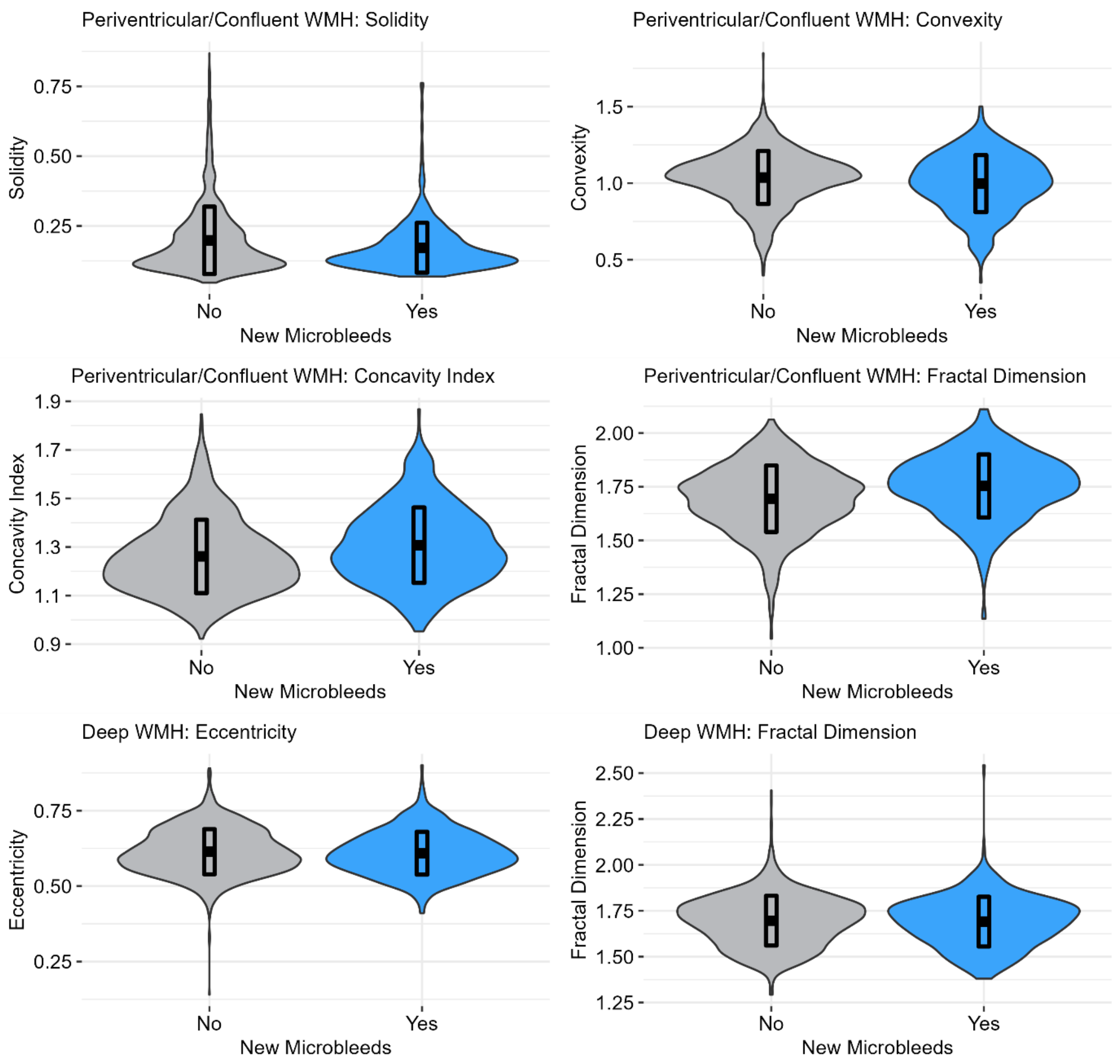

Supplementary figure 4. WMH shape markers at baseline in participants with new microbleeds versus participants without new microbleeds at follow-up. A more irregular shape of periventricular/confluent WMH at baseline was associated with new microbleeds (lower solidity (OR: 1.24 (1.07–1.44); p=0.004); lower convexity (OR: 1.16 (1.04–1.30); p=0.009); higher concavity index (OR: 1.24 (1.11–1.39); p<0.001); higher fractal dimension (OR: 1.47 (1.30–1.65); p<0.001)). A less elongated shape of deep WMH was associated with new microbleeds at follow-up (lower eccentricity: OR:1.14 (1.01–1.27); p=0.027).

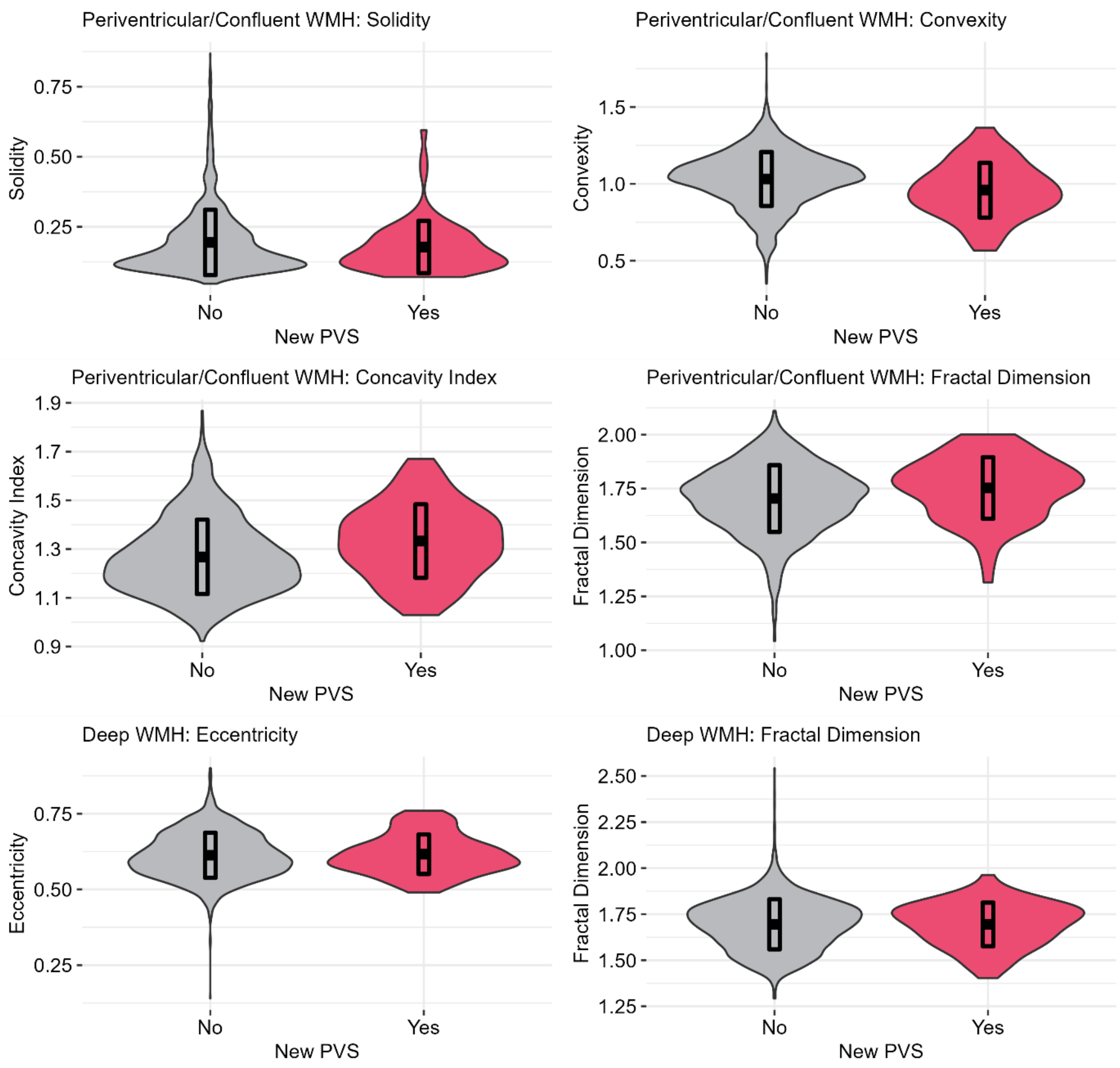

Supplementary figure 5. WMH shape markers at baseline in participants with new enlarged perivascular spaces versus participants without new enlarged perivascular spaces at follow-up. A more irregular shape of periventricular/confluent WMH at baseline was associated with new enlarged perivascular spaces (lower convexity (OR: 1.34 (1.05–1.71); $p<0.017$); higher concavity index (OR: 1.34 (1.05–1.71); $p<0.020$)). We did not find an association of deep WMH shape markers and new enlarged perivascular spaces.

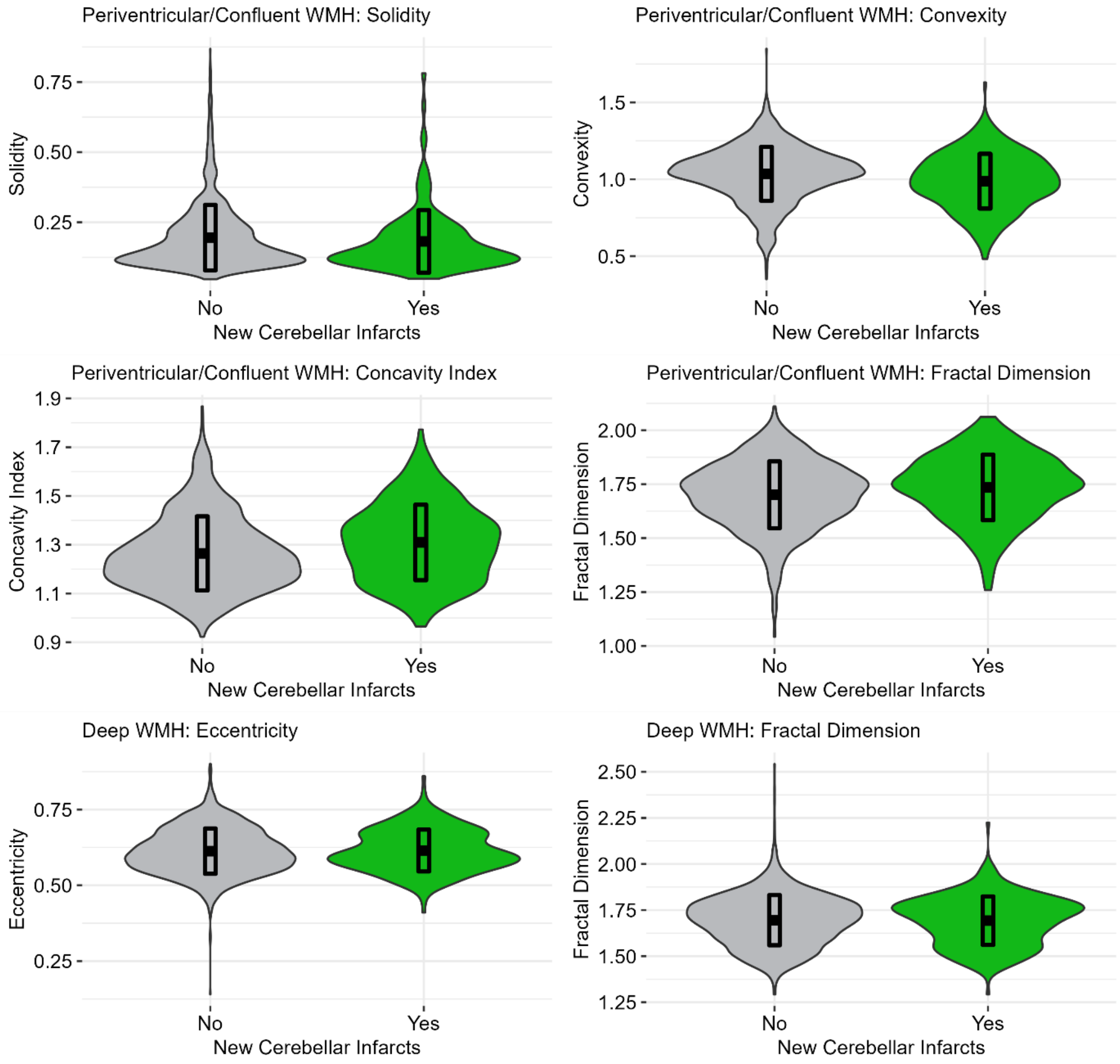

Supplementary figure 6. WMH shape markers at baseline in participants with new cerebellar infarcts versus participants without new cerebellar infarcts at follow-up. A more irregular shape of periventricular/confluent WMH at baseline was associated with new cerebellar infarcts at follow-up (lower convexity (OR: 1.16 (1.02–1.33); $p<0.022$); higher concavity index (OR: 1.16 (1.02–1.33); $p<0.027$)). We did not find an association of deep WMH shape markers and new cerebellar infarcts.

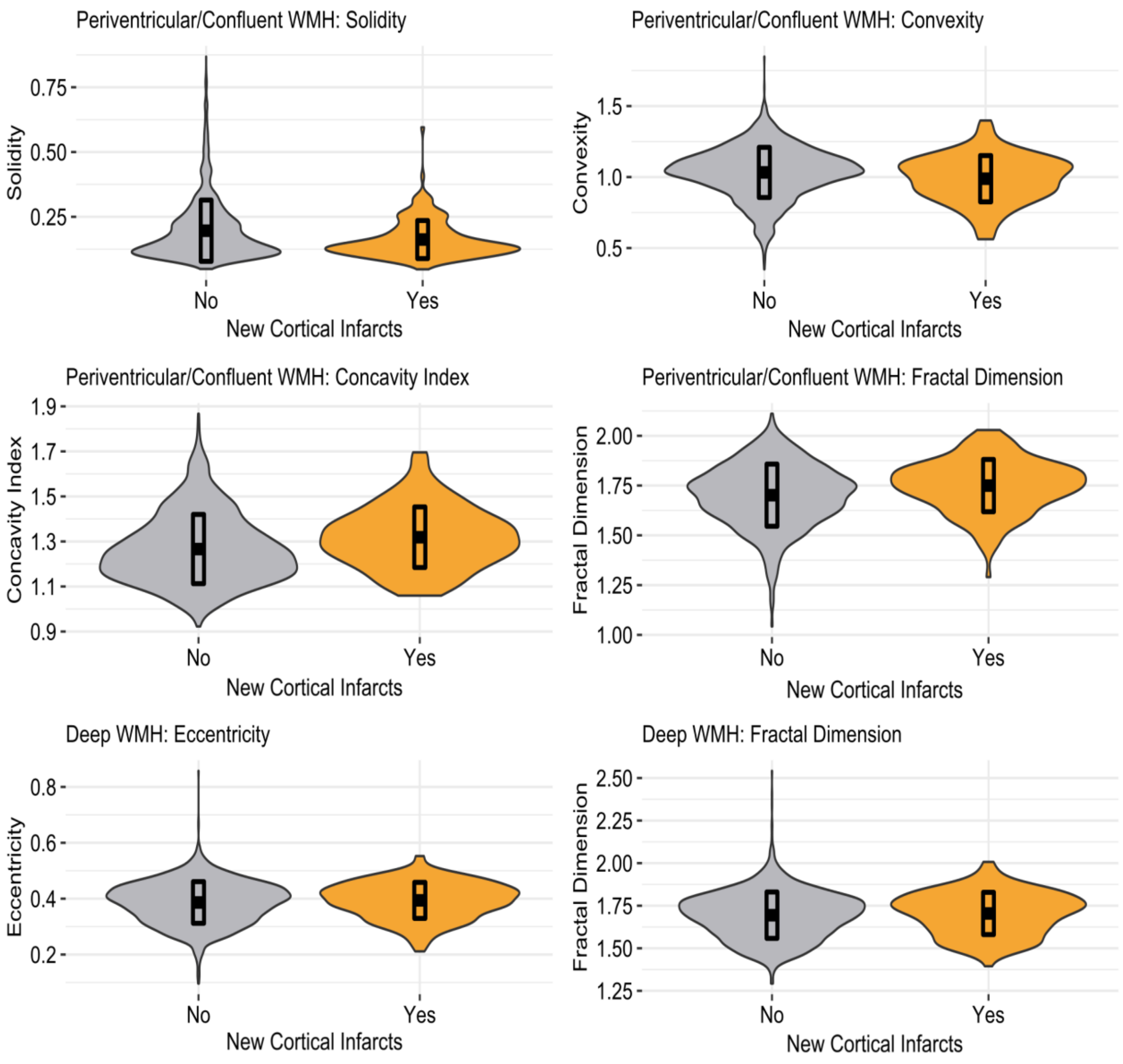

Supplementary figure 7. WMH shape markers at baseline in participants with new cortical infarcts versus participants without new cortical infarcts at follow-up. Periventricular/confluent WMH shape at baseline was not significantly associated with new cortical infarcts at follow-up. A less elongated and irregular shape of deep WMH at baseline was significantly associated with new cortical infarcts at follow-up (lower eccentricity (OR: 1.30 (1.10–1.55); p<0.003); higher fractal dimension (OR: 1.31 (1.11–1.55); p=0.001)).
